# IL-22 Is Produced by Innate Lymphoid Cells and Limits Inflammation in Allergic Airway Disease

**DOI:** 10.1371/journal.pone.0021799

**Published:** 2011-07-18

**Authors:** Christian Taube, Christine Tertilt, Gabor Gyülveszi, Nina Dehzad, Katharina Kreymborg, Kristin Schneeweiss, Erich Michel, Sebastian Reuter, Jean-Christophe Renauld, Danielle Arnold-Schild, Hansjörg Schild, Roland Buhl, Burkhard Becher

**Affiliations:** 1 III. Medical Clinic, Johannes Gutenberg-University, Mainz, Germany; 2 Institute of Immunology, Johannes Gutenberg-University, Mainz, Germany; 3 Department of Pediatrics, Johannes Gutenberg-University, Mainz, Germany; 4 Institute of Experimental Immunology, Department of Pathology, Neuroimmunology Divison, University Hospital Zurich, Zurich, Switzerland; 5 Institute for Molecular Biology and Biophysics, ETH Zurich, Zurich, Switzerland; 6 Ludwig Institute for Cancer Research Ltd Experimental Medicine Unit, Universite Catholique de Louvain, Louvain, Belgium; University Freiburg, Germany

## Abstract

Interleukin (IL)-22 is an effector cytokine, which acts primarily on epithelial cells in the skin, gut, liver and lung. Both pro- and anti-inflammatory properties have been reported for IL-22 depending on the tissue and disease model. In a murine model of allergic airway inflammation, we found that IL-22 is predominantly produced by innate lymphoid cells in the inflamed lungs, rather than TH cells. To determine the impact of IL-22 on airway inflammation, we used allergen-sensitized IL-22-deficient mice and found that they suffer from significantly higher airway hyperreactivity upon airway challenge. IL-22-deficiency led to increased eosinophil infiltration lymphocyte invasion and production of CCL17 (TARC), IL-5 and IL-13 in the lung. Mice treated with IL-22 before antigen challenge displayed reduced expression of CCL17 and IL-13 and significant amelioration of airway constriction and inflammation. We conclude that innate IL-22 limits airway inflammation, tissue damage and clinical decline in allergic lung disease.

## Introduction

Interleukin (IL)-22 belongs to a family of cytokines structurally related to IL-10 and was originally identified as a gene induced by IL-9 in T cells and mast cells [1]. The functional IL-22 receptor is composed of two subunits the IL-22R1 and the IL-10R2 chain, of which the latter can also pair with IL-10R1 to form the IL-10R complex [2], [3]. In contrast to IL-10, which performs predominantly regulatory functions during inflammation [4], IL-22 was initially identified as a pro-inflammatory cytokine capable of inducing the production of acute-phase reactants by hepatocytes [5]. *In vitro*, IL-22 has a pro-inflammatory, hyperplastic effect on keratinocytes [6], and it was reported that IL-22 mediates IL-23-induced dermal inflammation and acanthosis in mice [7], similar to the changes seen in psoriatic skin lesions in humans. On the other hand, Flavell and colleagues suggested that IL-22 can protect hepatocytes during acute liver inflammation [8]. Additionally, IL-22 has been shown to play a protective role in different models of inflammatory bowel disease [9]. IL-22 is expressed in healthy human lung tissue and decreased levels have been observed in patients with sarcoidosis and acute respiratory distress syndrome [10]. Exposure to IL-22 leads to an expression of host defense genes in humans and mice and neutralization of IL-22 resulted in exacerbation of bacterial infections, suggesting a protective role in mucosal/epithelial host defense [11]. In contrast, IL-22 plays only a marginal role for infection control during primary influenza virus infection in the lung [12]. Recent studies have shown increased pulmonary IL-22 production following different stimuli. Following bleomycin exposure IL-22 had either proinflammatory or tissue protective effects depending on the presence of IL-17A [13]. In contrast, IL-22 was protective during the development of lung fibrosis induced by chronic exposure to *Bacillus subtilis* [14]. Different sources have been described for IL-22 production. Following bleomycin increased numbers of IL-22 producing Th17 cells have been reported whereas following infection with *Bacillus subtilis* γδ T cells seem to be the major source of IL-22 production [13], [14]. In other organ systems, especially lymphoid tissue and the intestine, innate lymphoid cells have been recently described to be important producers of IL-22 [15]. Apart from NK cells and γδ T cells, the family of innate lymphoid cells is ever expanding and new nomenclature for these cells is being proposed [16]. IL-22-producing innate lymphoid cells share several common phenotypic and transcriptional similarities, but largely lack expression of most lineage markers [17]. Their role in lung disease is not well defined. Initial studies have suggested that in allergic airway disease IL-22 production is increased [18], however cellular source and functional role of IL-22 during the development for allergic airway disease are not identified.

Allergic asthma is characterized by airway inflammation, increased mucus production and airway hyperresponsiveness (AHR). Inflammation is orchestrated primarily by T helper (Th) 2 cells, which accumulate in the lung following allergen exposure and produce a vast array of different effector cytokines, including IL-4, IL-5, IL-13 and TNF [19], [20]. In addition to Th2 cells, the role and function of IL-17-secreting Th cells in allergic disease has lately become a subject of great interest. Increased levels of IL-17A and IL-17F have been reported in lungs of patients with severe asthma [21]. In murine models IL-17A is necessary during the development of sensitization to an allergen, but apparently functions as a negative regulator in established allergic airway disease [22]. To define the role of IL-22 in allergic responses within the lung, we applied a model of allergic asthma in mice sensitized to ovalbumin (OVA). We found significantly elevated levels of IL-22 in inflamed compared to non-inflamed lungs mainly produced by innate lymphoid cells, suggesting a so far unknown function of this cytokine. To determine the impact of IL-22 on lung inflammation, we used IL-22 deficient mice and discovered that IL-22 acts as a negative regulator for the development of allergic airway disease. Furthermore, we demonstrate that treatment of sensitized wild type mice with recombinant IL-22 before allergen exposure can reduce the development of AHR and airway inflammation, suggesting that exploiting this pathway could provide a potential therapeutic avenue for the treatment of allergic asthma.

## Results

### Allergen specific T cell responses result in increased expression of IL-22 in the lung

**Figure 1:**
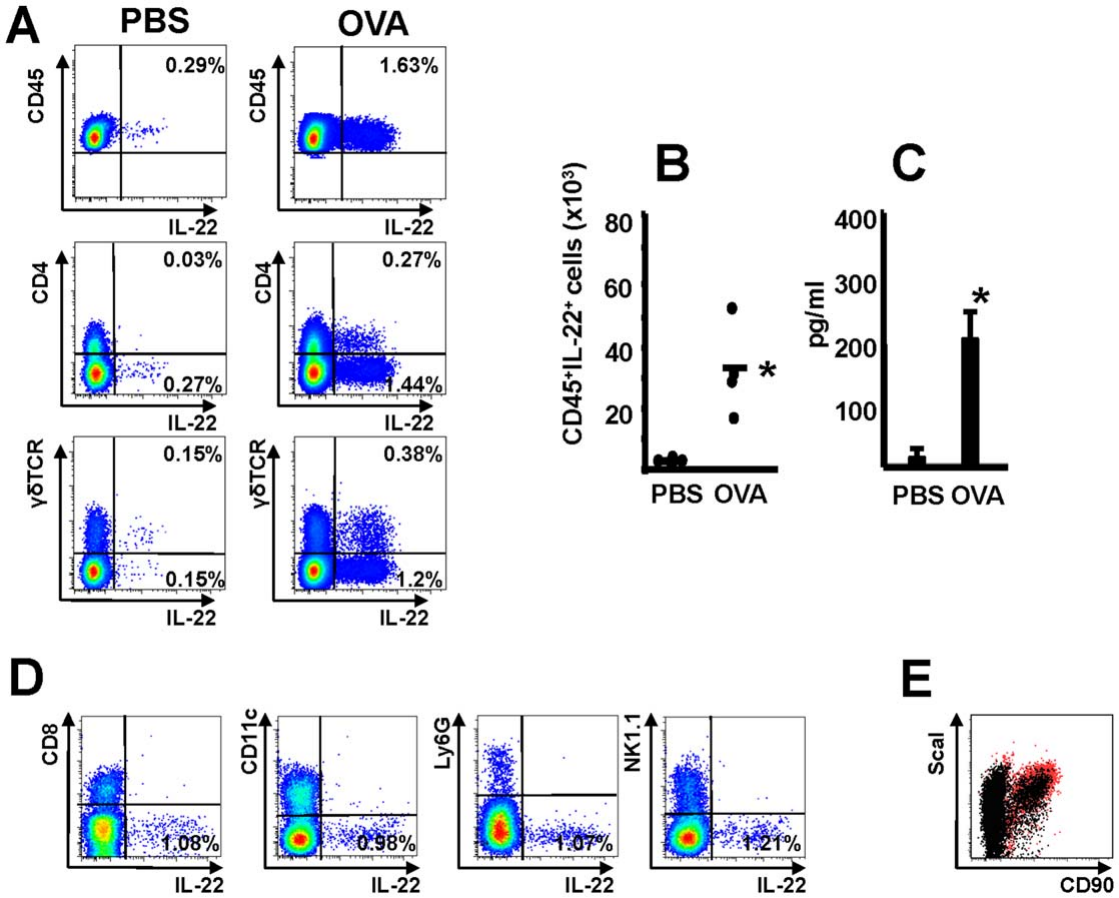
IL-22 expression is increased during specific T cell responses in the lung. Panel A: IL-22 intracellular staining in lung cells 24 hrs after inhaled exposure of OT II mice with either PBS (PBS) or OVA for 3 consecutive days. Panel A shows IL-22 a representative intracellular staining of CD45^+^ cells. Panel B: Numbers of CD45^+^ IL22^+^ cells from 2 independent experiments, each dot represents a single mouse, bar represents Mean, * p<0.05; Panel C: levels of IL-22 in BAL fluid, mean±SEM are shown, n = 4 from 2 independent experiments, Panel D: representative IL-22 intracellular staining in lung cells 24 hrs after inhaled exposure of OT II mice OVA for 3 consecutive days. Panel E: ScaI and CD90 expression in lung cells 24 hrs after inhaled exposure of OT II mice. Red dots represent IL-22 positive cells, black dots represent IL-22 negative cells.

To specifically assess whether IL-22 expression is altered during allergen specific T cell responses, mice which express transgenic T cell receptor for OVA_323–339_ (OT II) were exposed via the airways with OVA or PBS on 3 consecutive days. At 24 hrs following last challenge inflammatory cells in the lung were analyzed. OT II mice that received OVA showed increased levels of IL-22 by ELISA in BAL fluid (Figure 1C). Furthermore, FACS analysis revealed an increased number of IL-22-expressing cells in the lung tissue of OT II OVA treated mice compared to OT II mice which received only PBS (Figure 1 A, B). To delineate the population of IL-22-producing cells, next we took advantage of the combination of intracellular cytokine and cell surface staining. We used markers for a variety of adaptive/innate immune cells which we expected to release IL-22 in the lungs. Indeed, IL-22 producing cells were CD45 positive, however majority of IL-22 producing cells were lin-negative (Ly6G/C, CD4, CD8, CD11c, NK1.1, γδ TCR and Ly6G). Instead, they stained for CD90, Sca-1 (Figure 1E).

**Figure 2:**
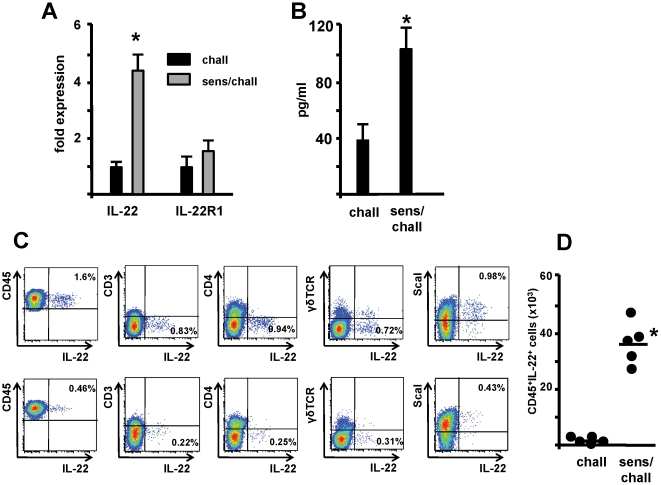
IL-22 expression is increased during allergic airway inflammation. Panel A: Expression of IL-22 and IL-22 R1 (IL-22 Rc) was assessed in lung tissue of challenged only (chall, n = 6) and sensitized and challenged (sens/chall, n = 6) animals. Total RNA was isolated 24 hours after the last challenge, reverse transcribed, and gene expression analyzed by PCR with specific primers for IL-22R1. Data are shown as fold induction relative to expression in naïve animals after normalization to GAPDH. Mean±SEM from 2 independent experiments are given. * p<0.05. Panel B: Levels of IL-22 in BAL fluid 48 hrs following the last challenge in challenged only (chall, n = 6) and sensitized and challenged (sens/chall, n = 6) animal. Mean±SEM from 2 independent experiments are given. * p<0.05. Panel C and D: IL-22 intracellular staining in lung cells 24 hrs following the last exposure in sensitized and challenged (top row) and challenged only (bottom row) animals and frequency of CD45^+^IL-22^+^ cells in lung tissue each dot represents a single mouse from 2 independent experiments. * p<0.05.

**Figure 3:**
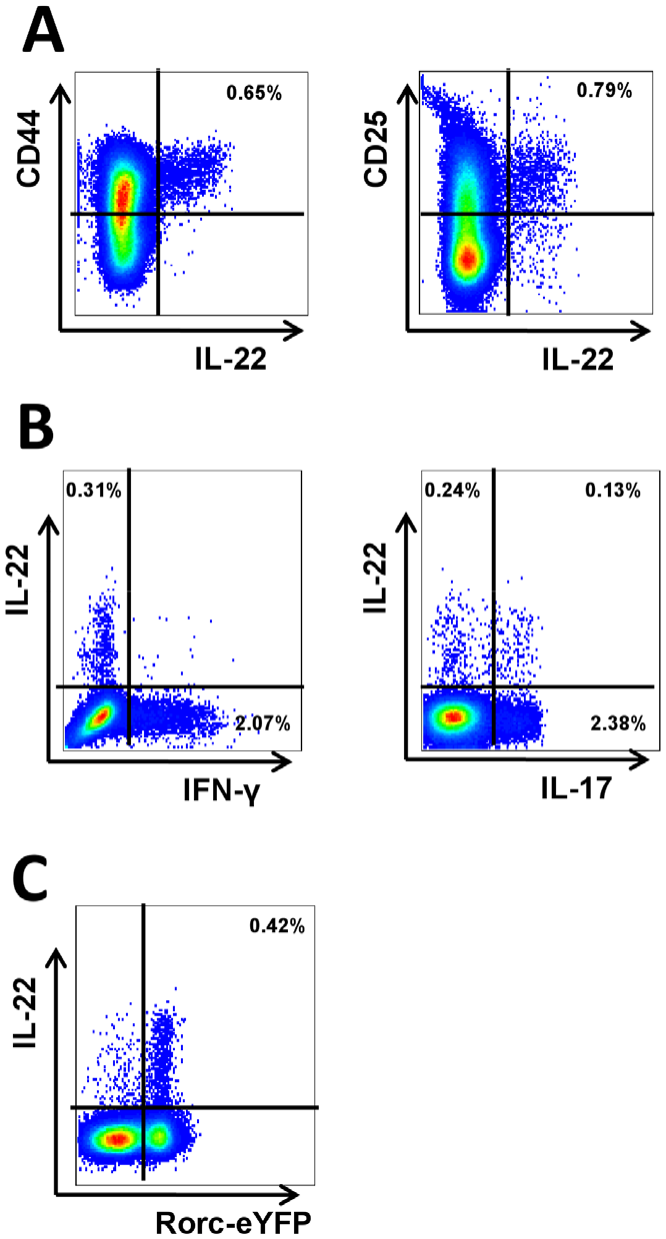
Analysis of cytokine production and surface markers of infiltrating mononuclear cells in the lungs. Panel A shows the expression of CD25 and CD44 among IL-22 producing cells. Panel B shows the IFN-γ and IL-17A production from sensitized and challenged mouse lungs. Panel C shows the expression of Rorgt. Rorc-eYFP mice were sensitized and challenged and the lung infiltrating mononuclear cells were analyzed for the expression of YFP.

To further assess if IL-22 expression also occurs in allergic airway disease, wt C57BL/6 mice were sensitized with OVA/Alum on day 0 and 14 and challenged with an OVA aerosol on days 28–30. Lungs were isolated from challenged-only, and sensitized and challenged mice and the expression of IL-22 and IL-22R1 was assessed. When compared to challenged-only mice animals, IL-22 expression was increased in the lung tissue of sensitized and challenged animals, whereas expression of IL-22R1 remained unchanged (Figure 2 A). In addition, increased levels of IL-22 were detected in BAL fluid of sensitized and challenged mice compared to challenged only animals (Figure 2 B). To further identify the source of IL-22 intracellular cytokine staining was performed on isolated lung cells. Interestingly, in our hands in sensitized and challenged wild type animals IL-22 secretion is still coming from this CD45^+^ IL-22-expressing innate lymphoid cell population and is drastically increased in the inflamed lungs following allergen challenge (Figure 2 C, D). Further analysis of these cells revealed that IL-22 producing cells express CD44 and CD25 (Figure 3A). These cells do not produce IFN-γ whereas some also express IL-17A (Figure 3B). Furthermore analysis of mice that express Cre recombinase in RORγt+ cells, which leads to terminal fate mapping of all cells which during their development expressed RORγt, showed that the vast majority of IL-22 positive cells are eYFP positive (Figure 3C).

### *Il22*-deficient mice show increased AHR and airway inflammation

**Figure 4:**
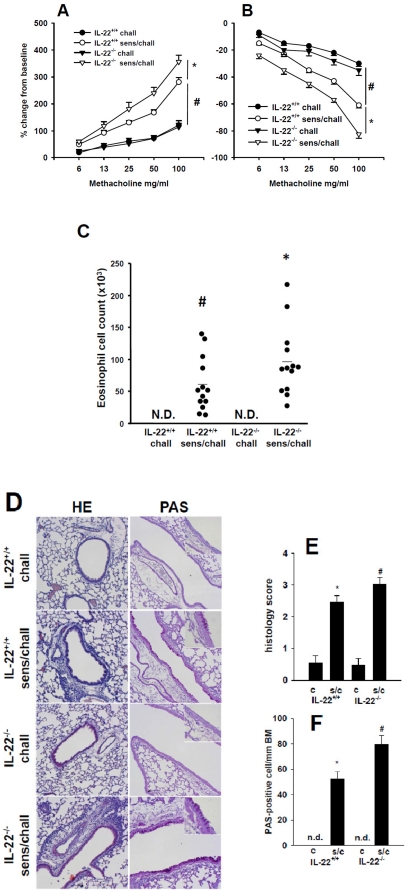
IL-22 deficient animals display increased AHR and airway inflammation. Airway resistance (panel A) and dynamic compliance (panel B) in challenged only wild-type (IL-22^+/+^ chall, n = 12), challenged only IL-22 deficient (IL-22^−/−^ chall, n = 12), sensitized and challenged wild-type (IL-22^+/+^ sens/chall, n = 13) and sensitized and challenged IL-22 deficient (IL-22^−/−^ sens/chall, n = 13) mice. Results are expressed as mean±SEM from 2 independent experiments. # p<0.01, *p<0.05. Panel C shows differential cell counts for eosinophils in BAL fluid. Each dot represents a single mouse, bar represents mean. # p<0.05 compared to IL-22^+/+^ chall and IL-22^−/−^ chall; * p<0.05 compared to all other groups. N.D.: not detectable. Panel D: Tissue inflammation was evaluated 48 hrs following the last challenge using hematoxylin and eosin staining (HE) and PAS staining for goblet cells in challenged only wild-type mice (IL-22^+/+^ chall), sensitized and challenge wild-type mice (IL-22^+/+^ sens/chall), challenged only IL-22 deficient mice (IL-22^−/−^ chall) and sensitized and challenged IL-22 deficient (IL-22^−/−^ sens/chall). Final magnifications 100× and 400× for inserts. Panel E shows histology score and panel F number of goblet cells per mm of basement membrane for challenged only (c) and sensitized and challenged (s/c) wild-type (IL-22^+/+^) and IL-22 deficient (IL-22^−/−^) animals. Each groups contains 12 animals. Means±SEM are given. *p<0.01 compared to IL-22^+/+^ c and IL-22^−/−^ c. # p<0.05 compared to all other groups.

Using genetically modified animals, which lack IL-22 expression, the role of IL-22 during the development of allergic airway disease was assessed. OVA-sensitized and non-sensitized *IL22^−/−^* mice and *IL22^+/+^* controls were challenged with an OVA aerosol. Airway reactivity was assessed by measuring the changes in airway resistance to increasing doses of inhaled methacholine (MCh) 48 h following the last airway challenge. Sensitized and challenged *Il22^−/−^* mice demonstrated a significantly (p<0.05) increased response to MCh throughout the dose–response curve compared to the sensitized and challenged *Il22^+/+^* mice (Figure 4 A, B). Under challenged-only conditions, no difference was observed between the *Il22^−/−^* and *Il22^+/+^* mice indicating that the protective IL-22 response is initiated during the sensitization rather than non-specifically after the acute mucosal exposure to OVA. Challenged-only *Il22^−/−^* mice showed a similar low responsiveness as challenged-only *Il22^+/+^* mice.

Inflammatory cell accumulation in the BAL fluid and lung tissue was evaluated 48 h after the last airway challenge. As expected, sensitized and challenged *Il22^+/+^* mice showed an increase in total cell counts compared with challenged-only mice. Importantly, sensitized and challenged *Il22^−/−^* mice showed clearly elevated total cell numbers in the BAL fluid when compared with sensitized and challenged wt mice (data not shown). We further assessed the differential cell count and could identify eosinophils to be markedly elevated (Figure 4 C). In accordance with the results observed in BAL fluid, sensitization and airway challenge of *Il22^−/−^* mice resulted in increased peribronchial inflammation observed in HE-stained sections of lung tissue (Figure 4 D, E). Inflammation was more pronounced in sensitized and challenged *Il22^−/−^* animals compared to sensitized and challenged *Il22^+/+^* animals. In line with the differences seen in inflammatory changes, also the number of PAS positive goblet cells was higher in the sensitized and challenged *Il22^−/−^* animals compared to the sensitized and challenged *Il22^+/+^* mice (Figure 4 D, F).

**Figure 5:**
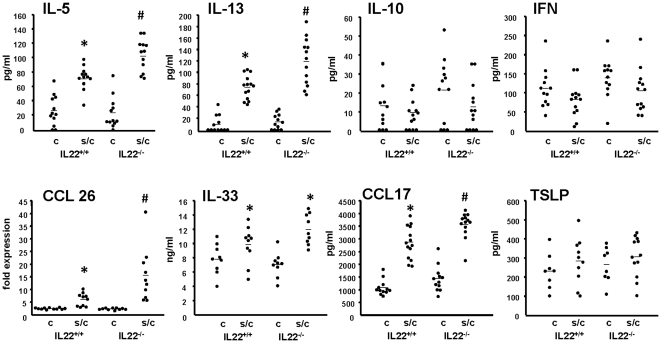
Analysis of cytokines and chemokines in IL-22^−/−^ and congenic controls. Levels of IL-5. IL-13, IL-10, IFN-γ (IFN) and CCL-17 in BAL fluid, TSLP and IL-33 in lung homogenate and expression of CCL26 in whole lung were analyzed in challenged only (c) and sensitized and challenged (s/c) *Il22* deficient (IL-22^−/−^ c, n = 12; IL-22^−/−^ s/c, n = 13) and congenic wild-type controls (IL-22^+/+^ c, n = 12; IL-22^+/+^ s/c, n = 13). Each dot represents a single mouse, bar represents mean. Data are from 2 independent experiments. * p<0.05 compared to challenged groups, # p<0.05 compared to all other groups.

**Figure 6:**
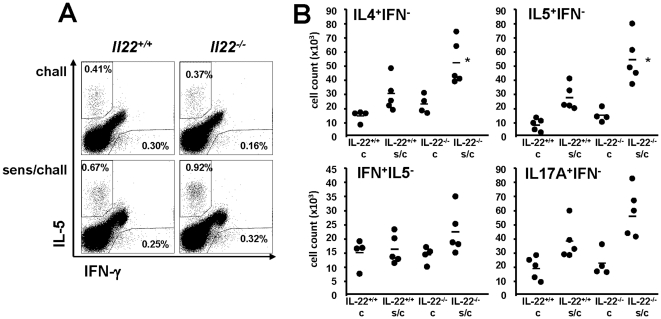
Intracellular cytokine staining of mononuclear cells in the lung. Cells were isolated from either challenged only (c) or sensitized and challenged (s/c) *Il22* deficient and congenic wild-type controls (IL-22^+/+^). Panel A shows a representative plot of intracellular IL-5 and IFN-γ staining. Panel B shows cell counts for IL-4 positive, IFN-γ negative (IL-4^+^IFN^−^), IL-5 positive, IFN-γ negative (IL-5^+^IFN^−^), IFN-γ positive, IL-5 negative (IFN^+^IL-5^−^) and IL-17A positive, IFN-γ negative (IL-17A^+^IFN^−^) cells. Each dot represent a single mouse. Data from 2 independent experiments are given. * p<0.05 compared to all other groups.

In addition, expression of different cytokines and chemokines were analyzed in BAL fluid and lung tissue. Sensitized and challenged *Il22^−/−^* animals showed increased levels of IL-5 and IL-13 in BAL fluid and increased expression of CCL26 (eotaxin 3), CCL17 (TARC) and IL-33 in lung tissue compared to sensitized and challenged *Il22^+/+^*. No differences between *Il22^−/−^* and *Il22^+/+^* animals were observed for IL-10, IFN-γ, and TSLP (Figure 5). Furthermore, the frequency of IL-4, IL-5, IFN-γ and IL-17A producing cells was measured in lung tissue. Increase numbers of IL-4^+^IFN-γ^−^, IL-5^+^IFN-γ^−^ and IL17A^+^IFN-γ^−^ cells were detectable in lung tissue in sensitized and challenged *Il22^−/−^* mice compared to sensitized and challenged *Il22^+/+^*animals, whereas no difference was observed for IFN-γ^+^ IL-5^−^ cells (Figure 6). Overall these results suggest a protective effect of IL-22 on the development of allergic airway disease as demonstrated by increased eosinophil numbers in BAL fluid, tissue inflammation and goblet cell metaplasia in *Il22^−/−^* mice compared to sensitized and challenged congenic wild-type animals.

### IL-22 does not affect systemic humoral immunity to neo-antigen

As inflammation in this model of allergic airway disease is characterized primarily by a Th2 response, which promotes humoral immunity, we sought to determine whether IL-22 might also contribute to the priming of anti-OVA humoral immunity. To assess this, we took serum of sensitized and challenged and challenged only mice 48 h after the last airway challenge and measured serum levels of OVA-specific IgE and IgG1 by ELISA. Sensitized and challenged *Il22^+/+^* mice showed increased levels of OVA-specific IgE and IgG1 compared with challenged-only control mice (Table 1). However, levels of total OVA-specific IgE and IgG1 in *Il22^−/−^* mice were found to not differ significantly between sensitized and challenged *Il22^+/+^* mice. Furthermore, allergen-induced proliferation and cytokine production of T cells was not different in sensitized and challenged *Il22^−/−^* mice compared to *Il22^+/+^* controls (data not shown). These results indicate that IL-22 is not necessary for priming of humoral immunity in this model, which is in line with our previous demonstration that IL-22 deficiency has no impact on Ag-driven lymphocyte priming and expansion [23].

**Table 1:** Serum immunoglobulin titers.

	*Il22^+/+^ chall*	*Il22^+/+^ sens/chall*	*Il22^−/−^ chall*	*Il22^−/−^ sens/chall*
OVA-specific IgE (Titer)	N.D.	287.4±44.6#	N.D.	307.1±37.9#
OVA-specific IgG1 (Titer) (×10^3^)	15.2±9	312.8±76.5#	32.2±25.1	349.3±110.3#
OVA-specific IgG2b (Titer)	6±5	560±216#	6±5	447±159#

Mice were sensitized and challenged as described in Methods. Serum levels of immunoglobulins were assessed 48 h after the last challenge. Mean values±SEM are given. *Il22^−/−^*: C57Bl/6 IL-22 deficient mice, *Il22^+/+^*: congenic wild-type control mice. N.D.: not detectable.

#p<0.05 compared to *Il22^+/+^*chall and *Il22^−/−^* chall.

### IL-22 induces STAT-3 phosphorylation in lung epithelial cells and reduces IL-13 and TNF induced chemokine production

**Figure 7:**
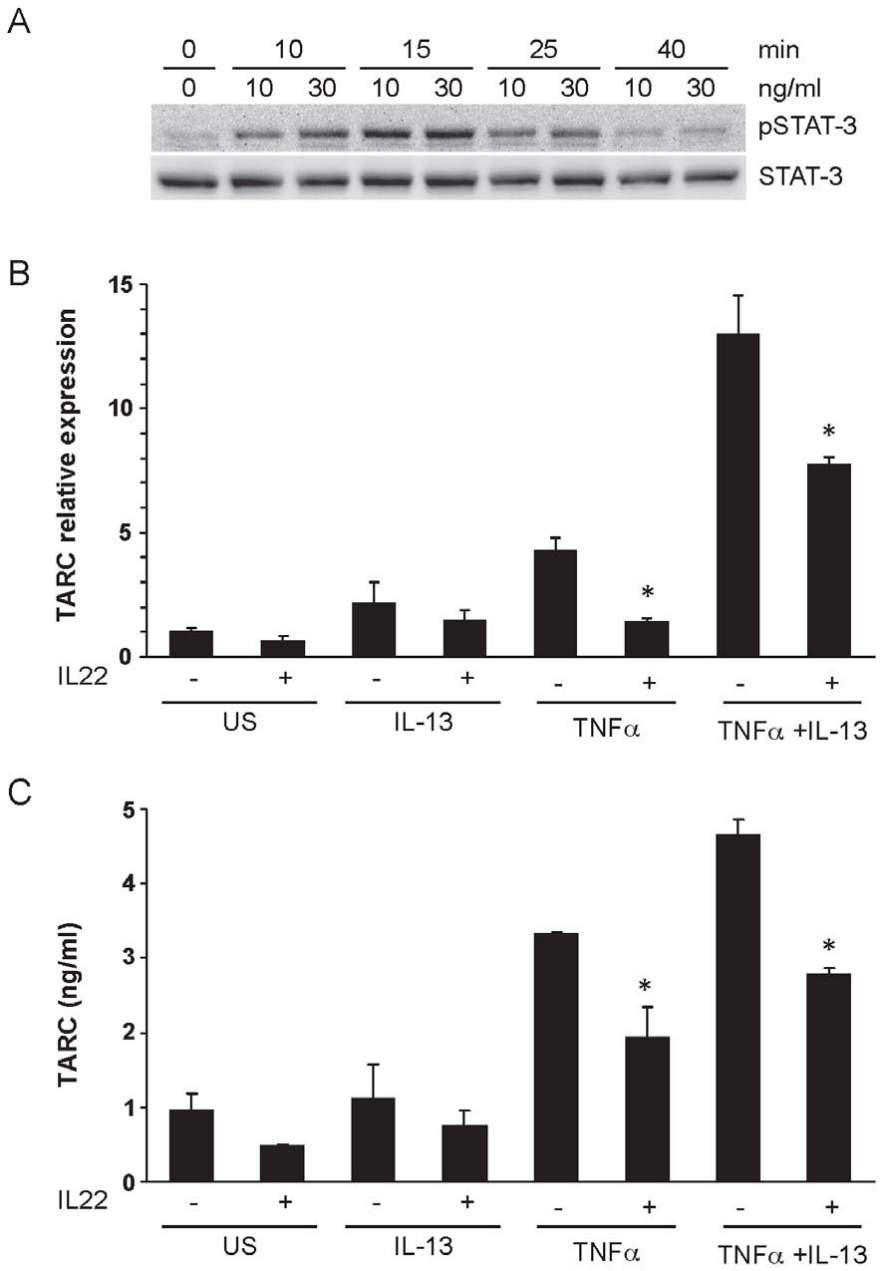
IL-22 reduces TNFα/IL-13 induced production of CCL17 in murine Clara cells. *Panel A:* C22 cells were stimulated with recombinant IL-22, for the indicated time points. Protein lysates were subjected to SDS page gel electrophoresis and Western blot analysis performed with a pSTAT-3 specific antibody. Detection of total STAT-3 as loading control. Data are representative of 2 independent experiments. *Panel B and C:* C22 cells were stimulated with recombinant IL-13, TNFα, or a combination of both, for 5 hours or left untreated (US). To assess the effect of IL-22 on TNFα and TNFα/IL-13 induced TARC expression, cells were preincubated with recombinant IL-22 for 4 hours and stimulated with the indicated cytokines for an additional 5 hours in the continued presence of IL-22 (IL-22 +). Controls were incubated with IL-22 only for a total of 9 hours. Relative TARC mRNA expression (panel B) was measured in triplicates by quantitative PCR and normalized to GAPDH expression levels. Results are shown as means±SEM of 5 replicates per treatment pooled from 4 independent experiments. TARC levels in cell culture supernatants (panel C) were assessed by ELISA after 48 hours. Results are shown as mean±SEM of triplicates and of 3 independent experiments. *p<0.01 vs. US, **p<0.001 vs. TNFα alone, ***p<0.05 vs. respective controls stimulated in the absence of IL-22.

As IL-22 is reported to act primarily on epithelial cells we assessed which cells in the lung may mediate the effect of IL-22, we analyzed by RT-PCR the expression of IL-22R1 and IL-10R2 in different cell types i.e. Th2 and Th17 cells, granulocytes, smooth muscle cells, dendritic cells and the immortalized murine clara cell line C22 as a model for bronchial epithelial cells (data not shown). Of all cell types analyzed, we could detect expression of the specific IL-22R1 chain only in C22 cells whereas IL-10R2 chain expression was found in all cell types analyzed. To further assess the mechanism by which IL-22 could limit airway inflammation, we analyzed the effect of IL-22 on C22 cells. As previously reported for various other cell lines [8], [24], [25] IL-22 stimulation led to increased STAT-3 phosphorylation also in C22 cells (Figure 7A). In order to investigate a potential modifying effect of IL-22 on clara cell function, C22 cells were pretreated with or without IL-22 and subsequently stimulated with IL-13, TNF, or a combination of both. Both cytokines are increased in allergic airway inflammation and have been shown to induce pro-inflammatory changes in bronchial epithelial cells. Based on the *in vivo* results, showing increased production of CCL17 in *Il22^−/−^* mice, we analyzed the generation of CCL17, which is an important chemokine for the attraction of CCR4 bearing Th2 cells [26] and the airway epithelium represents an important source of CCL17 [27]. While IL-13 alone did not significantly alter the expression of CCL17 by C22 cells (Figure 7B, C), we observed an increased expression of this chemokine upon TNF stimulation with a synergistic effect of both cytokines together. Interestingly, the induction of CCL17 could be significantly reduced by previous treatment of the cells with IL-22, suggesting that IL-22 reduces cytokine-induced chemokine production in these cells. Inhibition of CCL17 expression was indeed a specific IL-22-mediated effect as it could be reversed by pre-incubation of IL-22 with both a neutralizing IL-22-specific mAb and recombinant IL-22 binding protein (data not shown). In contrast, IL-22 alone had no significant effect on expression of CCL17 by C22 cells.

### Administration of rIL-22 reduces the development of allergic airway disease in sensitized and challenged mice

**Figure 8:**
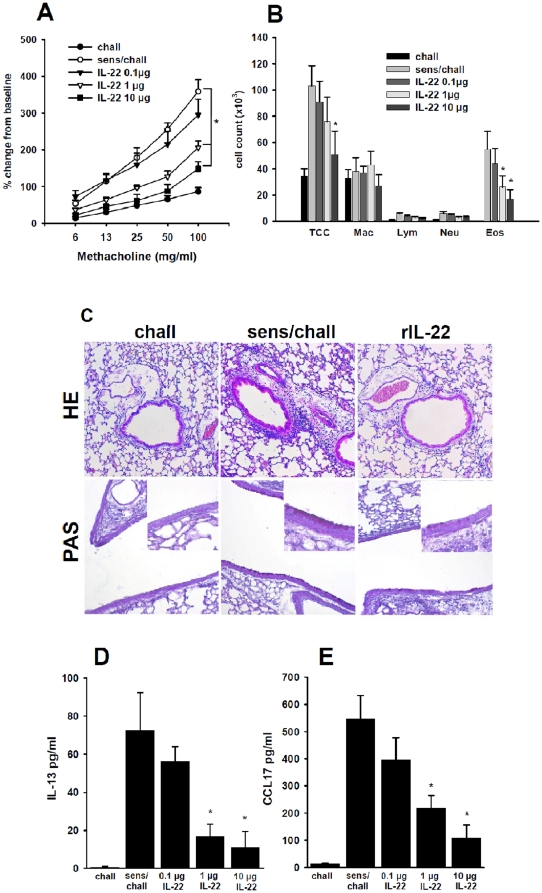
Administration of rIL-22 reduces AHR and airway inflammation. Airway responsiveness (panel A), cell counts in BAL fluid (panel B) and lung tissue inflammation and goblet cell metaplasia (panel C) were assessed in mice 48 h after the last airway challenge. Mice which were sensitized and challenged (sens/chall, n = 12) showed increased airway reactivity and numbers of eosinophils in BAL fluid compared to challenged only mice (chall, n = 5), where no eosinophils were detectable. Intranasal treatment of sensitized and challenge animals with 0.1 µg recombinant IL-22 (IL-22 0.1 µg, n = 12) showed little effects on AHR and inflammation. In contrast, mice treated with either 1 µg (IL-22 1 µg, n = 12) or 10 µg (IL-22 1 µg, n = 12) of recombinant IL-22 showed decreased AHR and number of eosinophils in BAL fluid. Means±SEM are given, *p<0.05 compared to sens/chall. *Panel C:* Tissue inflammation was evaluated 48 hrs following the last challenge using hematoxylin and eosin staining (HE) and PAS staining for goblet cells in challenged only mice (chall), non-treated sensitized and challenged mice (sens/chall) and sensitized and challenged animals treated with 10 µg of recombinant IL-22 (rIL-22). Final magnifications 100× and 400× for inserts. *Panels D and E:* Levels of IL-13 (panel D) and CCL17 (panel E) were measured in BAL fluid by ELISA 48 h after the last challenge. Means±SEM of challenged only mice (chall, *n* = 5), non-treated sensitized and challenged mice (sens/chall, n = 12), and sensitized and challenged mice treated with 0.1 µg (0.1 µg IL-22, n = 12), 1 µg (1 µg IL-22, n = 12) and 10 µg (10 µg IL-22, n = 12) of rIl-22, respectively. Mean±SEM are given. * p<0.05 compared to sens/chall and 0.1 µg IL-22.

To further establish the function of IL-22 in the development of allergic airway disease, we determined whether the therapeutic application of IL-22 is effective in wildtype mice. Recombinant IL-22 (rIL-22) was administered (intranasally) in different dosages after sensitization, but before challenge to C57BL/6 mice. To rule out any effect of rIL-22 independent of the inflammatory setting, we administrated rIL-22 to non-sensitized animals and confirmed that it had no impact on airway reactivity per se (data not shown). Overall direct effects of IL-22 on smooth muscle cells are unlikely as murine smooth muscle cells do not express the IL-22R1 (data not shown). However, administration of rIL-22 to sensitized mice before each challenge resulted in a significant reduction of AHR compared to the sham-treated controls (Figure 8 A). Furthermore, this IL-22 mediated AHR suppression was dose dependent as it was more pronounced in mice which received 10 µg rIL-22 prior to the challenges than in mice which received 1 µg.

To assess whether treatment with rIL-22 also reduced airway inflammation in this model of allergic airway disease, we evaluated the number and composition of infiltrating cells in BAL fluid. Sensitized and challenged animals which received rIL-22 showed clearly decreased numbers of eosinophils compared to sensitized and challenged control animals in a dose dependent manner (Figure 8 B). Similarly, peribronchial inflammation was reduced in mice treated with 1 µg and 10 µg, respectively, compared to the non-treated controls (Figure 8 C; Table 2). Also the number of goblet cells was reduced in sensitized and challenged animals treated with rIl-22 compared to sensitized and challenged controls (Table 2).

**Table 2:** Histology score and PAS-positive cells in airway epithelium.

	*chall PBS*	*sens/chall PBS*	*sens/chall 0.1 µg IL-22*	*sens/chall 1 µg IL-22*	*sens/chal 10 µg IL-22*
Histology score	0.5±0.3	2.9±0.4	2.8±0.7	2±0.4*	1.2±0.5*
PAS-positive cells/mm BM	N.D.	63±11	60±12	44±12*	19±9*

Peribronchial inflammation was graded by a semi-quantitative score (no inflammation = 0 to severe inflammation = 4). For each slide 5 randomly chosen areas were scored. Goblet cell metaplasia is expressed as number of PAS-positive cells per mm of basement membrane (BM). Mean values±SEM are given, n = 12 per group. N.D.: not detectable.

*p<0.05 compared to sens/chall PBS.

To determine whether rIL-22 treatment also resulted in an alteration of cytokines and chemokines present in BAL, we measured the concentrations of IL-13 and CCL17 in sensitized and challenged mice 48 h after the last challenge. Levels of CCL17 were strongly increased by OVA sensitization and challenge. We observed decrease in BAL CCL17 levels upon prior treatment with rIL-22 in a dose dependent manner (Figure 8 E). Accordingly, IL-13, which is produced by Th2 cells and known to be a critical mediator of allergic asthma, was decreased by rIL-22 in a dose dependent manner (Figure 8 D). These results further confirm that IL-22 ameliorates AHR and airway inflammation and suggests that IL-22 treatment led to a decrease in Th2 cell recruitment and Th2 cytokine production.

## Discussion

The development of allergic airway disease includes complex interaction of innate and adaptive immune cells as well as structural cells which also play an important role during this process [28], [29]. A pivotal step during the induction of airway inflammation is the recruitment of allergen specific effector T cells into the lung, which in turn produce different effector cytokines [19], [30]–[32]. However, in animals with allergen-specific transgenic receptor we identify an additional population of lineage negative lymphocytes which accumulate in the lung following allergen challenge. Similarly, this cell population was also detected in increased numbers in wild-type animals. Again we found an increase of these lineage negative cells following airway challenge only in mice which were previously sensitized but not in non-sensitized animals. Similar to other groups we found these cells expressing CD25 and CD44 [33], [34], both markers indicating cellular activation. Fate-mapping using *Rorc*-eYFP mice, we found the IL-22-producing cells to be RorγT-positive innate lymphocytes.

Overall this cell phenotype is reminiscent of a population of innate lymphocytes, which lack markers of mature lymphoid cells yet bear receptors commonly found on lymphoid progenitors and which rely on the activity or the transcription factor Rorγt [35]. Different types of innate lymphocytes have been described [36]. Innate lymphocytes are found in the gut mucosa and are responsible for the development of chronic colitis in mice [33]. Also, innate lymphocytes have been identified in fat-associated lymphoid clusters [37] and in mesenteric lymph nodes of helminth-infected mice [38], [39]. In the adult human small intestine, both innate lymphocyte population and an additional type of lymphocytes that is phenotypically similar to natural killer cells but lack cytotoxic function, express Rorγt and secrete IL-22 [15], [40], [41]. In regard to pulmonary inflammation mainly NKT and γδ T cells have been in the focus of research [42]. Indeed, mice deficient of NKT cells fail to develop AHR [43] and either proinflammatory or suppressive functions have been associated with the expression of either Vγ1^+^ or Vγ4^+^ TCR respectively [44], [45]. However, we found increased numbers of innate lymphocyte as the main source of IL-22 production during the development of allergen specific airway inflammation in the lung. In previous studies using different models, increased expression of IL-22 and accumulation of CD4^+^ IL-22^+^ cells have been reported following installation of bleomycin or chronic infection with *Bacillus subtilis* in the lung [13], [14]. Different sources have been described for IL-22 production. Following bleomycin increased numbers of IL-22 producing Th17 cells have been reported whereas following infection with *Bacillus subtilis* γδ T cells seem to be the major source of IL-22 production [13], [14]. In the present study the majority of IL-22 positive staining cells in the lungs were innate lymphocytes and only few CD4+ or γδ T cells were detected to express IL-22. In addition, IL-22 production on a per cell level was rather small. However, a substantial increase in the number of IL-22 producing cells was detectable in sensitized and challenged animals. Overall these findings suggest that in the present model IL-22 production is not induced but rather the recruitment of IL-22-producing innate lymphocytes into the lung is increased in sensitized and challenged animals.

Increased expression of IL-22 was detected in intracellular staining, real time PCR and by ELISA in BAL fluid in OT II mice that received OVA as well as allergen sensitized and challenged animals. This is in line with findings of increased IL-22 levels in lung homogenates of sensitized and challenged BALB/c mice [18]. Using genetically modified animals, deficiency of IL-22 resulted in increased AHR, eosinophilic airway inflammation, goblet cell metaplaisa, accumulation of IL-4 and IL-5 producing cells in the lung as well as increased levels of IL-5, IL-13, CCL17 and CCL26 in BAL fluid and lung tissue compared to challenged only mice. Under challenged-only conditions, no difference was observed between the *Il22^−/−^* and *Il22^+/+^* mice indicating that the protective IL-22 response is initiated during the sensitization rather than non-specifically after the acute mucosal exposure to OVA. Analysis of allergen specific antibodies indicate that that IL-22 is not necessary for priming of humoral immunity in this model, which is in line with our previous demonstration that IL-22 deficiency has no impact on Ag-driven lymphocyte priming and expansion [23].

Effects of IL-22 in lung has been reported on airway epithelia cells as only epithelial cells have been found to express IL-22 receptors in the lung. Previous reports have suggested a direct effect of IL-22 on pulmonary dendritic cells [18], however we were not able to find expression of IL-22 receptor in bone marrow derived dendritic cells as well as dendritic cells isolated from the lung. Also we did not find a direct effect of IL-22 on bone marrow derived dendritic in regard to STAT-3 phosphorylation, allergen-uptake and expression of co-stimulatory molecules (data not shown). In order to investigate a potential modifying effect of IL-22 on bronchial epithelial cells we utilized a clara cell. C22 cells were pretreated with or without IL-22 and subsequently stimulated with IL-13, TNF, or a combination of both. Both cytokines are increased in allergic airway inflammation and have been shown to induce pro-inflammatory changes in bronchial epithelial cells. T cell recruitment into the lung during allergic airway disease has been linked to chemokines, including CCL17 [26]. Based on the *in vivo* results, showing increased production of CCL17 in *Il22^−/−^* mice, we analyzed the generation of CCL17, which is an important chemokine for the attraction of CCR4 bearing Th2 cells and the airway epithelium represents an important source of CCL17 [27]. We observed an increased expression of this chemokine upon TNF stimulation with a synergistic effect of both cytokines together. Interestingly, the induction of CCL17 could be significantly reduced by previous treatment of the cells with IL-22, suggesting that IL-22 reduces cytokine-induced chemokine production in these cells. These findings support the in vivo data of increased levels of CCL17 in the lungs of sensitized and challenged *Il22^−/−^* mice. Also this is in line with a previous report, which demonstrated that IL-22 alone and in synergy with IL-10 decreased IL-8 production by human alveolar epithelial cell lines [10] and comparable to effects of IL-22 alone on bronchial epithelial cells observed in other studies [11].

These *in vitro* findings are also supported by *in vivo* studies of rIL-22 into the lung of sensitized mice prior to airway challenge. Indeed, rIL-22 suppressed AHR, airway inflammation and levels of IL-13 and CCL17 in the BAL fluid in a dose dependent manner demonstrating a suppressive capacity of IL-22. These findings therefore support the data generated with gene-deficient animals. As IL-22-deficient mice showed significant but small increase in AHR, the exogenous application of IL-22 had a very profound effect. These could suggest that IL-22-producing innate lymphoid cells play only a minor role as regulators during the development of AHR but that therapeutic application of IL-22 have a very beneficial effect. Overall the results suggests that IL-22 treatment led to a decrease in Th2 cell recruitment and Th2 cytokine production, primarily via inhibiting production of chemokines by epithelial cells In regards to the therapeutic value of this pathway IL-22 might offer the opportunity to specifically affect tissue responses without systemic effects when administered locally [46]. Treatment with IL-22 using gene delivery provided protection during experimental hepatitis [47]. Treatment of already inflamed colonic tissue with local gene-delivery of IL-22 lead to reduced inflammatory infiltrates and increased number of goblet cells in the gut [48]. In the lung IL-22 seems to play a dual role depending on the existing mircomilieu. Indeed, in bleomycin induced lung injury models *Il22^−/−^* animals show amelioration of disease, whereas IL-22 seems to plays a protective role in the same model in *Il17A*- deficient mice [13] whereas in chronic infection with *Bacillus subtilis* IL-22 seems to be tissue protective [14]. In the present study, based on the data in IL-22-deficient mice and administration of rIL-22, we found that IL-22 functions as a negative regulator in allergen-induced development of AHR, lung eosinophilia, and goblet cell metaplasia upon allergen exposure of the sensitized host. The fact that treatment with rIL-22 is effective in reducing AHR and airway inflammation, even after sensitization, is intriguing, as it could be a novel therapeutic approach for patients with allergic asthma.

## Materials and Methods

### Mice

C57BL/6 mice were bred in the Zentrale Tierzuchtanstalt of the Johannes-Gutenberg-University Medical Center. C57BL/6 OVA TCR transgenic OTII, *Il22* deficient (*Il22* ^−/−^), and respective C57BL/6 (*Il22^+/+^*) mice were kept and bred at the University of Zürich. In some experiemtns *Rorc*-Cre [49] crossed with *Rosa26*-stop-eYFP mice [50] (called ‘*Rorc-*eYFP mice’ here) [51] (kindly provided by A. Diefenbach, Freiburg, Germany) express Cre recombinase in RORγt+ cells, which leads to excision of the stop cassette and consequent expression of enhanced yellow fluorescent protein (eYFP) driven by the *Rosa26* promoter. Mice were used at the age of 8–12 weeks. All animal procedures were conducted in accordance with current institutional guidelines and performed according to the Helsinki convention for the use and care of animals and the Cantonal Veterinary Office of Zurich and were reviewed and approved by the review board of the Cantonal Veterinary Office of Zurich (ID: Z-BECH-Allg.) and the regional government authorities of Rhineland-Palatinate (ID: 23177-07/G08-1-016).

### Experimental protocol

OTII mice were anesthetized (Ketamin-ratiopharm®/Rompun 2%) (Ratiopharm, Ulm/Bayer, Leverkusen) and challenged intranasally with 20 µg ovalbumin (OVA, Sigma-Aldrich, St. Louis, MO) suspended in 20 µl PBS on 3 consecutive days. At 24 hours after the third allergen challenge, animals were sacrificed and lungs were dissected into small pieces and exposed to an enzymatic digestion by 0.5 mg/ml collagenase type IA (Sigma-Aldrich, Cat.# C9891) and a single-cell suspension was produced as previously described [52]. Mononuclear cells were cultured in RPMI (Invitrogen, Basel, Switzerland) with 10% FCS in the presence of 40 µg/ml ovalbumin (OVA grade V, Sigma-Aldrich, St. Louis, MO) overnight at 37°C and 5% CO_2_. After 12 h of culture PMA and Iono and Golgi plug (BD Biosciences, San Diego, CA, USA) were added for 5 h. Cell were then stained and analyzed by FACs (CantoII, BD Biosciences).

Wild-type and *Il22* ^−/−^ mice were sensitized by intraperitoneal (i.p.) injection of 20 µg OVA (Sigma-Aldrich) suspended in 2.25 mg aluminum hydroxide (Imject Alum, Pierce, Rockford, IL) in a total volume of 100 µl on days 0 and 14. Mice were then challenged via the airways on days 28, 29 and 30, using nebulized OVA (1% in phosphate buffered saline PBS), with an ultrasonic nebuliser (NE-U17, Omron, Hoofdorp, The Netherlands).

In some experiments, either 0.1, 1 or 10 µg of recombinant IL-22 was administered 1 h before each OVA challenge to sensitized C57BL/6 mice by intranasal application during general anesthesia (Ketamin-ratiopharm®/Rompun 2%) (Ratiopharm, Ulm/Bayer, Leverkusen). Each experiment was performed at least twice.

### Cloning, expression, and purification of murine interleukin-22

Murine IL-22 (34–179) was amplified from IL-23 stimulated splenocytes with GGAATTCCATATGCTGCCCGTCAACACCCG and CGCGGATCCTTAGACGCAAGCATTTCTCAGAGAC (Microsynth, Switzerland) forward and reverse oligonucleotide primers, respectively, and inserted into pIVEX2.4 (Roche) using the NdeI and BamHI (NEB) restriction sites. IL-22 was produced in a 20 ml batch mode cell-free protein expression reaction for 2.5 hours at 30°C and isolated. Insoluble material of the cell-free protein expression reaction was removed by centrifugation for 5 min at 4000×g. The supernatant was applied onto a 5 ml SP FF cation exchange column (Amersham) equilibrated with buffer A (50 mM sodium phosphate, pH 7.5) and proteins were eluted with 100 ml of a 0–1 M linear gradient of sodium chloride in buffer A. Fractions containing IL-22 were pooled and loaded onto a 5 ml HisTrap column (Amersham) equilibrated with buffer B (50 mM sodium phosphate, 30 mM imidazole, 500 sodium chloride, pH 7.5). IL-22 was eluted with 100 ml of a linear gradient of 30–500 mM imidazole in buffer B and the obtained protein was dialyzed overnight at 4°C in an 8 kDa Biotech RC membrane (Spectrum Labs) against 2 l of 1.5×PBS, pH 7.2, and 5% glycerol. The protein was then dialyzed four times against 2 l of 1×PBS with buffer exchange after every 2 h, followed by sample concentration in a 10-kDa Amicon Ultra-15 centricon (Millipore) at 4°C and 3500×g to obtain 70 µM IL-22 in 2 ml final volume. The sample was lyophilized in 250 µl aliquots and stored at −80°C until further use. Protein integrity was validated by gel filtration analysis, CD-, fluorescence-, and NMR-spectroscopy (data not shown). Content of LPS was measured by LAL kinetic chromogenic assay (Lonza, Wuppertal, Germany). The amount of LPS was <0.02 EU/µg protein.

### Measurement of airway reactivity

Measurements of the airway resistance (R_L_) and dynamic compliance (C_dyn_) were performed on anesthetized, intubated and mechanically ventilated mice (FlexiVent, Scireq, Montreal, QC) in response to increasing doses of inhaled methacholine (MCh) (6.25, 12.5, 25, 50 and 100 mg/ml) as previously described [53].

### Broncho-alveolar lavage and lung histology

After assessment of airway function, lungs were lavaged via the tracheal tube with 1 ml PBS. Numbers of BAL cells were counted by using trypan blue dye exclusion. Differential cell counts were made from cytocentrifuged preparations fixed and stained with the Microscopy Hemacolor ®-Set (Merck, Darmstadt, Germany). Percentage and absolute numbers of each cell type were calculated. Then lungs were fixed by inflation (1 ml) and immersion in 10% formalin and embedded in paraffin. Tissue sections were stained with hematoxylin and eosin (HE) and periodic acid-Schiff (PAS). Slides were examined in blinded fashion by 2 experienced observers with a microscope (BX40, Olympus, Hamburg, Germany). Peribronchial inflammation and number of goblet cells were assessed as previously described [52].

### Analysis of leukocyte populations by FACS

Mononuclear cells were stained with CD45 PerCP 30-F11 (Biolegend, San Diego, CA, USA), CD4 GK1.5 PE, FITC (BD Biosciences), CD4 Pacific blue RM4-5 (Biolegend), CD3 PE-Cy7 17A2 (Biolegend), CD3 Pacific blue 145-2C44 (BD Biosciences), CD90.2 Pacific blue 30-H12 (Biolegend), CD90.2 biotin 30-H12 (BD Biosciences), ScaI PerCP Cy5.5 D7 (Biolegend), TCRγδ FITC and PE GL3 (BD Biosciences), GR1 Pacific blue RB6-8C5 (Biolegend), CD8 Pe-Cy7 53-6.7 (BD Biosciences), CD25 PE PC61 (BD Biosciences), CD44 FITC IM7 (BD Biosciences), CD11c PE HL3 (BD Biosciences), NK1.1 PE PK136 (BD Biosciences), Ly.6G PE 1A8 (BD Biosciences) and IL-22 Alexa-647 3F11 provided by Genentech (San Francisco, CA, USA).

For intracellular cytokine staining mononuclear cells were incubated with PMA (Phorbol 12 Myristate 13 Acetate; SIGMA, Saint Louis, USA) and Inonomycin (SIGMA, Saint Louis, USA) and BrefeldinA (SIGMA, Saint Louis, USA) for 4–6 h at 37°C. Following incubation cells were washed twice and the intracellular staining was performed (BD Cytofix/Cytoperm) according to the manufactures protocol. In brief, Fc-receptor blocking antibodies (αCD16/CD32 BD Pharmingen, Heidelberg Germany) were added to the cells and incubated for 10 min at 4°C. For surface staining surface antibodies were added to the appropriate groups. To analyze T cells, CD4-FITC (BD Pharmingen); CD3-Percp Cy5.5 (BD Pharmingen) were used. Antibodies were incubated for 20 min at 4°C in the dark. Following washing in WB cells were resuspended in 150 µl Cytofix/Cytoperm (BD Pharmingen) and incubated for 20 min at 4°C in the dark. Cells were washed twice and subsequently intra-cellular antibodies were added. For analysis following antibodies were used: IFNγ-APC; IL17A-Alexa Fluor 647; IL17-PE, IL4-PE; IL4-APC and IL5-PE (all BD Pharmingen). Antibodies were incubated at 4°C for 30–40 min in the dark. Afterwards cells were washed twice, resuspended and then analyzed (Canto II, BD Pharmingen)

### Cytokine and antigen-specific immunoglobulin ELISA

IL-22 (Antigenix America, Huntington Station, NY, USA), IL-5, IL-10, IFN-γ (all BD Bioscience, San Diego, CA, USA) IL-13, IL-33, CCL17 and TSLP (all R&D Systems, Minneapolis, MN, USA), ELISAs were performed according to the manufacturer's directions. OVA specific IgG1 and IgE-titres in sera were determined as previously described [53].

### Cell culture and stimulation

The murine clara cell line C22 generated from H-2Kb-tsA58 transgenic mice was a kind gift of J. Ryerse and D. Demello (St. Louis, USA). The cells express the thermolabile large tumor antigen of the tsA58 strain of the simian virus 40 (SV40) under control of the interferon (IFN) γ inducible H2Kb promoter [54]. For experiments, C22 cells were plated in 6-well plates (Falcon) at a density of 0.75×10^6^ cells/well for purification of total protein and RNA, respectively, or in 96-well plates at a density of 2.5×10^4^ cells/well for ELISA, and cultured over night in non-permissive conditions, i.e. in media without IFNγ at 39°C. Cells were stimulated with 20 ng/ml or 60 ng/ml rmIL-13 (R&D Systems), 5 ng/ml TNFα (R&D Systems), and pretreated with rmIL-22 as indicated. C22 cells were maintained in Dulbecco's modified Eagle's medium DMEM (Gibco) supplemented with 3% FCS (Vitromex), penicillin (250000 U/ml), streptomycin (250 mg/ml), glutamine (2 mM), amphothericin B (250 µg/ml), endothelin-1 (200 ng/µl), insulin (10 µg/µl), transferrin (10 mg/ml), endothelium cell growth supplement (15 µg/µl), epidermal cell growth factor (0.1 µg/µl), hydrocortisone (5 µg/µl) and T3 (200 µg/ml) at 33°C at 10% CO_2_. For propagation, cells were kept at 33°C with supplementation of interferon γ (100 units/µl); for experiments, media without interferon y was used and cells were kept at 39°C.

### RNA Isolation, conventional and quantitative Real time PCR

For expression analysis of CCL17/TARC, IL-22, IL-22R1, and IL-10R2, total RNA was isolated from cells and total lung tissue, respectively, using TRIzol reagent (Invitrogen) and cDNA was synthesized with RevertAid M-MuLV reverse transcriptase (MBI Fermentas) following the manufacturer's recommendations.

Conventional PCR was performed on a Cycler Peqstar 96 Universal (Peqlab) with Platinum polymerase (Invitrogen) according to the manufacturer's instructions. qRT-PCR was performed on an iCycler (Bio-Rad, Munich, Germany) in triplicates using ABsolute SYBR Green Mix (ABgene, Hamburg, Germany) according to the manufacturer's instructions.

GAPDH forward: CCA TCA CCA TCT TCC AGG AG; GAPDH reverse: TTT CTC GTG GTT CAC ACC C; mCCL17 forward: AGA CAG GCA GAA GGA CCC ATG AAG, mCCL17 reverse: TAA TCC AGG CAG CAC TCT CGG C; mIL-22 forward, CCA GCC TTG CAG ATA ACA AC, mIL-22 reverse, GGA AGG AGC AGT TCT TCG T, IL-22R1 forward, ACA GCT GCC CTG CTT CTT AT, IL-22R1 reverse, ATT TGG GAG TGG AGA GGA TG. After normalization of the data according to the expression of GAPDH mRNA, the relative expression levels were calculated. Data were analyzed by the Δ/Δ^CT^.

### Western-Blot

After treatment of C22 cells with rmIL-22, cells of a confluent well of a 6-well plate were lysed in 250 µl 1×RIPA-buffer containing inhibitors for proteinases and phosphatases. Thirty µg protein were separated by denaturing SDS gel electrophoresis, and transferred to an Immobilon-P Membrane. After blocking in PBS/5% nonfat dry milk the membrane was incubated with a polyclonal rabbit antibody specific for STAT3 phosphorylated at Ser727, followed by incubation with an HRP coupled goat anti-rabbit antibody. Blots were developed using SuperSignal West Dura chemiluminescent substrate kit and visualized on a BioRad Molecular Imager ChemiDoc XRS System. To control for equal loading, blots were stripped, blocked and incubated with a rabbit polyclonal antibody recognizing total STAT3/β-Actin.

### Statistical analysis

ANOVA was used to determine the levels of difference between all groups. Differences in responsiveness to MCh were assessed by repeated measures ANOVA. Pairwise comparisons were then performed employing either Tukey-Kramer honest significant difference test or Bonferrini correction. Number of eosinophils, histology score and number of PAS-positive cells were initially analyzed by non-parametric ANOVA (Kruskal-Wallis Test) for overall differences. This was verified by pair wise comparisons (Mann-Whitney-U-Test). *P* values for significance were set at 0.05. Values for all measurements are expressed as the mean±SEM.
